# The use of biosimilar medicines in oncology - position statement of the Brazilian Society of Clinical Oncology (SBOC)

**DOI:** 10.1590/1414-431X20177214

**Published:** 2018-01-11

**Authors:** G.S. Fernandes, C. Sternberg, G. Lopes, R. Chammas, M.A.C. Gifoni, R.A. Gil, D.V. Araujo

**Affiliations:** 1 Sociedade Brasileira de Oncologia Clínica, Hospital Sírio-Libanês, São Paulo, SP, Brasil; 2 Sociedade Brasileira de Oncologia Clínica, Programa de Pós-Graduação em Anatomia Patológica, Faculdade de Medicina, Universidade Federal do Rio de Janeiro, Rio de Janeiro, RJ, Brasil; 3 Global Oncology Program, Sylvester Comprehensive Cancer Center, University of Miami, Miami, FL, USA; 4 Instituto do Câncer do Estado de São Paulo, Faculdade de Medicina, Universidade de São Paulo, São Paulo, SP, Brasil; 5 Fujiday Oncologia D’Or, Oncocentro, Hospital São Carlos, Fortaleza, CE, Brasil; 6 Instituto Nacional do Câncer, Oncoclínica, Rio de Janeiro, RJ, Brasil; 7 Departamento de Medicina Interna, Universidade Estadual do Rio de Janeiro, Rio de Janeiro, RJ, Brasil

**Keywords:** Immunobiologicals, Therapeutic antibodies, Biosimilar, Cancer management, Access

## Abstract

A biosimilar is a biologic product that is similar to a reference biopharmaceutical product, the manufacturing process of which hinders the ability to identically replicate the structure of the original product, and therefore, it cannot be described as an absolute equivalent of the original medication. The currently available technology does not allow for an accurate copy of complex molecules, but it does allow the replication of similar molecules with the same activity. As biosimilars are about to be introduced in oncology practice, these must be evaluated through evidence-based medicine. This manuscript is a position paper, where the Brazilian Society of Clinical Oncology (SBOC) aims to describe pertinent issues regarding the approval and use of biosimilars in oncology. As a working group on behalf of SBOC, we discuss aspects related to definition, labeling/nomenclature, extrapolation, interchangeability, switching, automatic substitution, clinical standards on safety and efficacy, and the potential impact on financial burden in healthcare. We take a stand in favor of the introduction of biosimilars, as they offer a viable, safe, and cost-effective alternative to the biopharmaceutical products currently used in cancer. We hope this document can provide valuable information to support therapeutic decisions that maximize the clinical benefit for the thousands of cancer patients in Brazil and can contribute to expedite the introduction of this new drug class in clinical practice. We expect the conveyed information to serve as a basis for further discussion in Latin America, this being the first position paper issued by a Latin American Oncology Society.

## Introduction

Since the approval of recombinant insulin – the first biopharmaceutical (biological product) approved by the US Food and Drug Administration (FDA) in 1982 – over 160 biological drugs and vaccines have been licensed in the United States alone.

Unlike simple drugs composed of small molecules, whose structure and characteristics are well-defined, biopharmaceuticals are derived from the application of biotechnology to industrial manufacturing of active substances from cells and/or microorganisms that are genetically manipulated to produce the drug. Currently, most biopharmaceuticals are produced in mammalian cells, because the post-translational modifications occurring in non-human mammalian proteins resemble those in human cells.

Biopharmaceuticals are structurally complex biological molecules that can be composed of nucleic acids, proteins, sugars or complex combinations of these substances. Protein biopharmaceuticals such as monoclonal antibodies (mAbs) can weigh up to 150,000 Daltons. Because of their complexity and the fact that the production process fundamentally influences the characteristics of the end-product, it is common and acceptable for biopharmaceuticals to have small detectable differences between batches, provided such heterogeneity does not affect the efficacy and safety of the end-product.

It takes on average 12 years for a biopharmaceutical to be developed, from discovery to registration. To obtain approval and registration, a biopharmaceutical must pass through three phases of research and development. The discovery phase takes 2–5 years and consists of the identification of substances with potential biological activity. The pre-clinical phase takes 1–2 years and involves *in vitro* studies to establish product manufacturing and formulation processes, testing to better understand the mechanism of action of the compound, development of analytical methods for quality control, and *in vivo* studies to establish efficacy and safety parameters. The clinical phase involves testing in humans to determine the drug’s safety, dosage, and efficacy parameters and establish treatment protocols. The clinical phase actually includes three phases (I, II, and III). Phase I clinical trials are designed to assess the safety of a drug in humans. Phase II trials are performed on larger groups and are designed to demonstrate clinical efficacy and drug safety. Phase III studies are designed to assess the effectiveness of a new compound in comparison with the current ‘gold standard’ treatment. Clinical trials are conducted only after they have received approval from health authority and ethics committees in the country where the drug is being developed. In Brazil, these studies are conducted in accordance with the guidelines of the National Committee for Ethics in Research (CONEP) and approval is granted by the National Health Surveillance Agency (ANVISA).

Developing a new biopharmaceutical is a long, costly, and high-risk process, and fewer than 10% of all compounds tested reach the market. The cost of developing a new drug – from *in vitro* studies to phase III clinical trials, and the associated risks – is added to the final cost of the marketed biopharmaceutical and is estimated to be approximately US$2 billion (1). In Brazil, biopharmaceuticals account for only 2% of all medications purchased by the Ministry of Health (MS), the other 98% being chemical-based drugs. However, biopharmaceuticals account for 41% of the MS drug budget (2).

There are currently 28 commercially available monoclonal antibody biopharmaceuticals for cancer treatment with 217 therapeutic indications. Non-Hodgkin’s lymphoma (NHL) has the largest number of indications for monoclonal antibodies, and rituximab – used mainly in the treatment of NHL – is the biopharmaceutical with the largest number of biosimilars developed or under development (3).

The costs of treatment incurred by cancer patients, their families, and public and private healthcare providers are prohibitive. The high cost of biopharmaceuticals, especially ‘monoclonal molecules’ like rituximab, account for most of the cost of cancer treatment. However, the introduction of monoclonal antibodies into healthcare practice has significantly improved the overall survival of cancer patients, while in general being less cytotoxic and having milder side effects compared to chemotherapeutic agents, and delivering precise target-directed treatment. One such drug is trastuzumab, which inhibits the expression of human epidermal growth factor receptor (HER2). When used in combination with chemotherapy, trastuzumab was associated with a significantly longer time to disease progression and improved overall survival in patients with breast cancer that overexpressed HER2 (4).

By 2019, the patents for some of the major biopharmaceutical products will have expired, opening up a huge opportunity for the development of similar biological medicinal products, the so-called biosimilars, which could compete with or even replace the biological drugs currently available in the market. In 2015, the FDA published a document with considerations on biosimilar development and the requirements for their approval; similar documents have been published in the European Union (EU) (5,6). A large number of biosimilars for nine biopharmaceuticals, three of which for cancer treatment (bevacizumab, trastuzumab, rituximab), are currently under development by different manufacturers in different countries. Over 1,000 monoclonal antibodies for cancer are currently under investigation, illustrating the great potential of the market (3).

In Brazil, public health is a constitutional right. Thus, the Brazilian government is the largest buyer of biological products in the country, accounting for 60% of all purchases made, raising the interest of national industries for the production of biological products or biosimilars. Currently, two national pharmaceutical consortia – Bionovis and Orygen – encompassing a range of national laboratories are involved in the development of biosimilars.

The Brazilian Society of Clinical Oncology (SBOC) actively participates in the discussion about the development and use of biosimilars in the country and acknowledges the need to share its recommendations and educate oncologists and other healthcare practitioners about the introduction of this new type of drug in clinical practice. In this document, we address a range of issues pertaining to biosimilar development and application in Brazil. Acting in the best interests of patient health and welfare, SBOC makes some recommendations for the appropriate use of this new class of therapeutics.

## Biosimilars

A biosimilar is a biologic product that is similar to a reference biopharmaceutical product. Biosimilars are not identical copies of biopharmaceuticals because it is not possible to copy a complex molecule with the technology currently available, but only reproduce a similar molecule with the same activity of the reference molecule. Thus, the concept of biosimilars is different to that of generic drugs, because the latter is an actual replicate of the structure and characteristics of the molecules of the reference drug. Table 1 presents the main differences between generic and biosimilar medicines.

**Table 1.** Comparison of generic and biosimilar medications.

Generics	Biosimilars
Simple chemical structure	Complex biological molecule
Single entity	Homogeneous mixture
Can be fully characterized	Cannot be fully characterized
Low potential for immune reactions	High potential for immune reactions
70% lower cost than reference product	15% lower cost than reference product

The biosimilar molecule must have an amino acid sequence, or primary structure, similar to that of the reference biopharmaceutical. However, while the patent for the biopharmaceutical may disclose some characteristics of the reference molecule such as molecular weight, sequence, and mode of action, follow-on manufacturers do not have access to detailed information about its production process. For instance, information on culture conditions, bioreactor system and design, filtration process, and centrifugation and purification techniques is not disclosed by the patent owner. Thus, the manufacturing process of a biosimilar is often different from that of the reference biopharmaceutical, resulting in differences in the end-product – including immunogenicity pattern, biological activity, and glycosylation of the molecule – which could affect the quality, safety, and efficacy of the biosimilar, and modify its properties in relation to the reference biopharmaceutical.

The development process of a biosimilar begins with pharmacokinetics and pharmacodynamics studies – there is no discovery phase, which can take up to 5 years. In the following phases, the biosimilar must demonstrate similar biological activity to the reference product. Preclinical analyses involve the use of up-to-date analytical tools with adequate sensitivity to detect even small differences in non-protein residues, quaternary structure, immunogenicity, and pharmacokinetic and pharmacodynamic properties (even for mAbs). Thus, the need for animal studies in demonstrating biosimilarity has recently been discussed by regulators (7). The alternative is to focus on producing sufficient evidence of similarities in chemical composition and biologic activity, through rigorous preclinical analytical validation, followed by safety and efficacy studies in patients (clinical phases) so that the safety data can be the same used for the reference biopharmaceutical (8,9). A clinical trial comparing the biosimilar and the reference biopharmaceutical is still required through either equivalence (recommended) or non-inferiority testing, provided that the similarity has been demonstrated with a comfortable margin of confidence.

Biosimilars can be developed using newer lower-cost technology than the one used for developing the reference product. Thus, the cost of manufacturing a biosimilar may be lower compared to the reference biopharmaceutical. However, unlike with generic drugs (>75% cost reduction) and because of their complexity, cost reductions for biosimilars range between 10–35%.

Fiprima® (filgrastim), a medication that is used to stimulate the proliferation and differentiation of granulocytes, is the first biosimilar developed in Brazil, approved by ANVISA in 2015 (10).

## Biosimilar regulation in Brazil

The pharmaceutical industry in Brazil still lacks the technology for the production of biosimilar oncology drugs, but public laboratories such as Bio-Manguinhos of the Oswaldo Cruz Institute (IOC) have signed technology transfer agreements and produced infliximab and interferon-gamma. However, according to ANVISA, there is great interest from private companies in acquiring the technology to produce oncology biopharmaceuticals and biosimilars.

To date, only two biosimilars have been approved in Brazil, but the number of biosimilar applications filed with ANVISA is expected to grow in the coming years.

The Brazilian regulations for the approval of biosimilars were discussed and enacted by ANVISA in 2010 – Collegiate Board Resolution (RDC) No. 55. In line with the world’s most respected regulatory authorities such as the FDA and the European Medicines Agency (EMA), the RDC has established that registration of a biopharmaceutical product may follow the pathway of an innovative drug or biosimilar. For the biosimilarity pathway, extensive preclinical documentation on the biosimilar characteristics in comparison to the reference product is provided, and the biosimilar must crucially demonstrate similarity to the reference product in terms of safety and efficacy based on clinical data. It is important to note that the clinical trial must be a comparative study with the reference medicine, and the outcome measures may range from drug outcomes (response rates or clinical benefit rates) to survival outcomes.

In Brazil, the package insert of a biosimilar product includes information about the reference product and, in some cases, may be the same as the one provided with the reference product. Data from comparative studies may be included in the biosimilar package insert. In addition, a biosimilar package insert includes basic information on biosimilars.

## Extrapolation of indications

Extrapolation is the approval of a biosimilar for use in all indications held by the reference biopharmaceutical other than the original indication for which the biosimilar had initially been developed and approved.

The medical sciences community and drug regulatory authorities around the world have not yet come to a consensus on the extrapolation of biosimilar indications. Nevertheless, there is a consensus that strict pharmacovigilance mechanisms are required to safely establish acceptable extrapolation practices.

Both the EMA and the FDA allow for the extrapolation of biosimilars on a case-by-case basis. In Canada, clinical data of the reference drug can be used for the biosimilar version. Japan and South Korea are flexible on the issue and formal requirements for extrapolation approval are less stringent in India than for the FDA or EMA (11).

In some cases, regulatory agencies may come to different decisions in allowing extrapolation of indications for a given biosimilar. CT-P13, the biosimilar to infliximab and the first biosimilar mAb, has only been tested for rheumatoid arthritis and ankylosing spondylitis (12). The extrapolation of indications has been granted for infliximab across more than 50 countries and the EU, but its approval for juvenile idiopathic arthritis, ulcerative colitis, and Crohn’s disease has been controversial (12,13).

The criteria for the approval of extrapolations involve testing that is sensitive enough to detect significant differences between the biosimilar and the reference biopharmaceutical. In addition, the mechanism of action and/or receptor involved in drug action must be the same for both biosimilar and reference drug and the safety and immunogenicity of the biosimilar must be thoroughly determined and characterized. The comparability study must demonstrate the bioequivalence of the drugs and a full clinical report must be submitted to ANVISA. The decision to allow extrapolation of indications for a given biosimilar is made by a panel of expert consultants advisory to the ANVISA.

SBOC believes that extrapolation for each proposed indication should ideally be supported by scientific evidence from a randomized phase III clinical trial. However, SBOC acknowledges that such studies are not always feasible and practical and may increase the costs and lengthen the approval process for new indications. SBOC recognizes that the extrapolation of indications has positive and negative arguments that should be weighed carefully by the regulatory authority. Arguments supporting extrapolation include the biological similarity between diseases, drugs that share the same therapeutic target (e.g., mAbs), testing in susceptible populations, and indications of the reference drug. Conversely, differences in immunogenicity, activation of biological pathways other than those associated with the reference medicine, and likely adverse effects of combination therapies are some of the arguments against automatic extrapolation. Thus, SBOC recommends that decisions regarding extrapolation should be made on a case-by-case basis, because current scientific evidence is not sufficient to automatically support extrapolation between the reference drug and its biosimilar(s).

## Interchangeability of medications

Interchangeability of a medicine refers to a situation where a medicine can be exchanged for another equivalent product with a proven equivalent efficacy and mode of action without the risk for an adverse health outcome.

Regulatory agencies have not come to a consensus on the interchangeability of reference products and biosimilars. In Europe, different countries have adopted similar interchangeability policies, whereas in the United States, even though FDA regulations permit interchangeable biosimilars to be substituted for the reference product, state laws may differ. In Canada, Health Canada does not accept interchangeability between biosimilars and their respective reference products.

The interchangeability of biosimilars is currently under discussion in Brazil and ANVISA allows it if the biosimilarity of a biosimilar with its reference product has been established based on clinical data obtained from studies that aimed to show the interchangeability between drugs. Patient follow-up and physician assessment are critical in determining whether a biosimilar can be considered interchangeable with the respective reference product.

However, as previously discussed by ANVISA, SBOC acknowledges that there are currently no clinical studies supporting the interchangeability of any biosimilars approved in Brazil with their respective reference products. SBOC supports that patients, as much as possible, should remain on the same biological medicine throughout treatment. Whenever this cannot be met, SBOC recommends that interchangeability should only occur under strict conditions, including the approval of the attending physician and without interference from the pharmacist. SBOC recommends that the switch from reference product to biosimilar should be discussed with the patient.

## Nomenclature

A debate over the naming system to be adopted for biosimilars is one of the main challenges for their acceptance. The World Health Organization (WHO) proposes that biosimilars of non-glycosylated compounds use the same international nonproprietary name (INN) as their reference product, whereas glycosylated biosimilars should receive a Greek letter suffix spelt in full as adopted in the EU (e.g., the epoetin biosimilar is named *epoetin zeta*). However, because biosimilars will outnumber their original reference products in the near future, the use of identical non-proprietary biosimilar names in prescriptions may lead to inaccuracies, making acceptance of biosimilars by the market harder. To date, no naming policy for biosimilars has been implemented in the United States.

SBOC warns of the importance of adopting a naming system that provides an ‘identifier’ to be used for all biosimilars, enabling pharmacovigilance at all times. Thus, it is essential that the physician be specific when prescribing biopharmaceutical medicines.

## Pharmacovigilance

Pharmacovigilance is the science and activities relating to the detection, collection, assessment, monitoring, and prevention of adverse effects with pharmaceutical products. Pharmacovigilance is instrumental in determining the incidence and severity of adverse events associated with a pharmaceutical product, which may ultimately lead to its withdrawal from the market.

Pharmacovigilance efforts are dependent not only on the accurate reporting of adverse events, but also the precise identification of the biosimilar that has been associated with such events. Thus, a standardized naming system is required to facilitate the identification of biosimilars and enhance product pharmacovigilance. In addition, it is important that the biosimilar be characterized with respect to its extrapolation, safety, and interchangeability and this information should be communicated to both physicians and pharmacists.

In the EU, a comprehensive risk management plan, including a plan for post-authorization safety surveillance, must be submitted to the authorities for any new biosimilar at the time of the marketing authorization (14). For the FDA, any risk assessment and risk mitigation strategies in place for the reference product are automatically included into the risk management program of the biosimilar. Nevertheless, in some cases, certain safety risks and the need for changing the indication of a biosimilar may be evaluated through post-marketing surveillance or clinical studies.

The case of epoetin illustrates the need for a cautious approach to the extrapolation of a drug’s indication. Epoetin was initially indicated to treat anemia in patients with cancer receiving concomitant myelosuppressive chemotherapy. It was later extrapolated to other indications, because some studies showed that its use promoted a potential survival benefit and improved tumor response in addition to increasing hemoglobin levels and eliminating requirements of red blood cell transfusion. However, the first clinical studies evaluating the use of epoetin in a wider range of indications in patients with cancer receiving chemotherapy suggested adverse effects on survival and time to tumor progression (15). The debate on the extrapolation of epoetin is presently ongoing.

SBOC recognizes the key role played by pharmacovigilance and warns of the importance of implementing a tracking system for biopharmaceutical and biosimilar products. In addition, SBOC recognizes that current pharmacovigilance programs in Brazil are insufficient and is of the opinion that cancer treatment centers across the country should adopt more stringent pharmacovigilance practices.

## Clinical trials

SBOC emphasizes the need to examine two classes of drugs in clinical oncology, the antineoplastic agents and the supportive drugs.

The acceptable outcomes in clinical trials will depend on each scenario and should be consistent with the disease and its stage. Even though survival outcomes are preferred in phase III trials, global regulatory agencies have accepted other endpoints such as pathological complete response and progression-free survival as sufficient to determine the safety and efficacy of biosimilars. It has generally been accepted that outcomes in biosimilar trials should be different from those expected for the reference product in oncology practice. For instance, the expected outcome for the trastuzumab biosimilar in neoadjuvant treatment of breast cancer is pathological complete response.

In Europe, clinical trials are required to demonstrate clinical comparability of biosimilars with their reference product, and the immunogenicity of the biosimilar must be assessed for different indications. In the United States, clinical studies or other studies are required to demonstrate safety, purity, and potency of the biosimilar in one or more appropriate conditions of use for which the reference product is licensed.

SBOC understands that clinical evidence should be provided for the approval of biosimilar oncology drugs. SBOC follows WHO recommendations that equivalence studies are preferred.

## Education

SBOC recognizes the urgent need to implement training on biosimilars and to introduce pharmacovigilance courses in medical schools and in the training of healthcare providers in the country, as well as training in proper reporting of medications and their association with adverse events. In addition, a cultural shift in the Brazilian medical community to the importance of reporting adverse events that may be associated with the use of biosimilars is crucial.

SBOC suggests that pharmacovigilance issues should be addressed in medical meetings and congresses, and discussions should be held to create a standalone database with information pertaining to the use of biosimilars.

SBOC suggests that biosimilar developers should play an active role in facilitating pharmacovigilance efforts.

## Economic impact of biosimilars

Cost-effectiveness analysis (CEA) measures the cost of an intervention in monetary units by the expected health gain in non-monetary units called natural units (e.g., years of life gained or recurrence-free survival). CEA provides an estimate of the incremental cost of the intervention per effectiveness unit. A health intervention is considered cost-effective when there is a justifiable clinical benefit for its cost.

The decision on whether the additional cost is justified by the additional effectiveness is made by society in the context of social values and limited resources. Even though the precise quantification of the acceptable cost for a given effectiveness (‘clinical benefit’) is difficult, health interventions that society chooses to incorporate represent valuable reference thresholds. The WHO recommends three times the gross domestic product *per capita* of the country where the analysis was found cost-effective and justifiable (16).

In biological therapy, a biosimilar medicine must demonstrate similarity to the biological reference product in terms of the physicochemical characteristics of the molecule, similarity in preclinical trials, and preferably, equivalence in clinical trials. In some cases, the demonstration of non-inferiority rather than equivalence in clinical trials is acceptable.

When biosimilarity is demonstrated, the clinical outcomes of both biosimilar and reference product should be similar, i.e., no incremental clinical benefit to the biosimilar exist. In this case, a cost-minimization analysis (CMA), which is an economic analysis used when two or more treatment alternatives have similar outcomes, is appropriate (17). CMA compares the cost of treatment when alternative therapies have demonstrated equivalent clinical effectiveness. Its goal is to identify the treatment alternative that can be provided for the lowest cost.

When biosimilarity is not demonstrated and clinical outcomes are different, cost-effectiveness analysis is recommended.

## Harmonization

Global harmonization for regulatory requirements is critical for the acceptance of biosimilars and facilitation of their development and approval, which may ultimately lead to significant cost reductions, not only for developers but also for final recipients.

The WHO published a guide outlining the general principles for the introduction of biosimilars, which serves as a basis for setting national requirements while respecting the realities of each local environment (18).

## Conclusion

In this document, SBOC takes a stand in favor of the introduction of biosimilars. It understands that biosimilars offer a viable, safe, cost-effective alternative to the biopharmaceutical products currently used in the treatment of several diseases, especially cancer. We hope this document can provide valuable information to support therapeutic decisions that maximize the clinical benefit for the thousands of cancer patients in Brazil and contribute to expedite the introduction of this new drug class in clinical practice.
